# Female genital tract microbiome: the influence of probiotics on assisted reproduction

**DOI:** 10.61622/rbgo/2024rbgo82

**Published:** 2024-12-04

**Authors:** Ana Sofia Almeida Santana, Ana Margarida Póvoa

**Affiliations:** 1 Universidade do Porto Faculty of Medicine Porto Portugal Faculty of Medicine, Universidade do Porto, Porto, Portugal.; 2 Centro Hospitalar Universitário São João Unit of Reproductive Medicine Department of Gynecology Porto Portugal Department of Gynecology, Unit of Reproductive Medicine, Centro Hospitalar Universitário São João, Porto, Portugal.; 3 Universidade do Porto Faculty of Medicine Department of Gynecology, Obstetrics and Pediatrics Porto Portugal Department of Gynecology, Obstetrics and Pediatrics, Faculty of Medicine, Universidade do Porto, Porto, Portugal.

**Keywords:** Pregnancy outcome, Infertility, female, Abortion, spontaneous, Pregnancy rate, Genitalia, female, Embryo implantation, Reproductive techniques, assisted, Microbiota, Probiotics

## Abstract

Assisted reproductive technology (ART) has been evolving since 1978, with the number of techniques performed increasing over the years. Despite continued advances, some couples continue to have difficulties getting pregnant, and it has recently been considered that the microbiome of the female genital tract (FGT) may influence embryo implantation and the establishment of pregnancy. This review aims to evaluate the role of probiotics on reproductive outcomes in infertile women on ART. A search throughout medical databases was performed, and six articles met the criteria. Five studies showed improvements in pregnancy rates, with only one demonstrating statistical significance. One article showed no improvement but reported a statistically significant reduction in the miscarriage rate in the probiotic group. Further research is needed to evaluate the true potential of probiotics, namely to assess whether they effectively modulate the FGT microbiome and if these changes are maintained over time.

## Introduction

Since the birth of the first child conceived through in vitro fertilization (IVF) in 1978, there has been a remarkable improvement in the success of assisted reproductive technology (ART), which is now practiced in more than a hundred countries around the world.^(1,2)^

As David Adamson, on behalf of International Committee for Monitoring Assisted Reproductive Technologies (ICMART), stated during the European Society of Human Reproduction and Embryology (ESHRE) 2023 annual meeting, at least 12 million babies were born through in vitro fertilization after the first one conceived, and the overall number of cycles is increasing by around 6.7% per year.^(3)^

According to the latest 23^rd^ ESHRE report, the number of ART cycles increased in 2019. Despite progress in fertility treatments, the implantation rate of transferred embryos remains low, with pregnancy rates per transfer standing only at 33.5% for ICSI, 34.6% for IVF, and 35.8% for FET, leading to delivery rates per transfer of 24.1%, 25.3% and 25.6%, respectively.^(4)^

Infertility represents a global health challenge, affecting approximately 48 million couples and 186 million people worldwide.^(5)^ Multiple factors have been identified to cause infertility such as age, ovarian reserve, and diseases. However, in some cases the cause of infertility remains unknown and recently, it has been considered that the woman's genital microbiota may also play an important role in embryo implantation and the establishment of pregnancy.^(6,7)^

The female genital tract (FGT) has its own microbiome.^(8)^ The healthy vaginal microbiome can be made up of more than 90% lactobacilli,^(9)^ while the upper tract is 100 to 1000 times less dense and has a greater variety of species.^(10,11)^ The vaginal microbiome comprises more than 50 species,^(12)^ the most prevalent being lactobacilli such as *L. iners, L. crispatus, L. gasseri, L. jenesenii,* followed by *L. acidophilus, L. fermentum, L. plantarum, L. brevis, L. casei, L. vaginalis, L. delbrueckii, L. salivarius, L. reuteri,* and *L. rhamnosus.*^(13)^ Lactobacilli are believed to play a crucial role in maintaining vaginal health by inhibiting the growth of pathogens like *Gardnerella vaginalis* through the production of bacteriocins, hydrogen peroxide, and lactic acid, thereby fostering an acidic vaginal pH (pH < 4.5).^(14–17)^ Moreno et al.^(9)^ reported a significant improvement in implantation, pregnancy, and live birth rates in infertile patients undergoing IVF, in Lactobacillus-dominant microbiota.^(9)^

On the other hand, a reduction in the dominance of lactobacilli favors vaginal dysbiosis, such as bacterial vaginosis, which is associated with the proliferation of anaerobic microorganisms that adhere to the vaginal epithelium, such as *Gardnerella vaginalis, Atopobium vaginae, Prevotella spp,* and *Veillonella spp.*^(10,18,19)^ Bacterial vaginosis is mostly asymptomatic, making it difficult to diagnose.^(20)^ However, it induces significant inflammatory responses that can influence a woman's reproductive capacity, potentially leading to conditions like endometriosis, infertility, miscarriages, and preterm births.^(21–24)^ Studies analysing the genital microbiome of infertile women have revealed a lower presence of lactobacilli and a higher incidence of bacterial vaginosis.^(25)^ Furthermore, a systematic review by van Oostrum et al.^(20)^ found that bacterial vaginosis significantly increases the risk of preclinical pregnancy loss, with an odds ratio of 2.36.^(20)^

Consequently, it has been speculated that manipulating the female genital tract microbiome could restore healthy vaginal flora, prevent vaginal dysbiosis and potentially improve reproductive outcomes in infertile women, which could translate into an increase in implantation and pregnancy rates and a reduction in miscarriage rates.^(10,26)^ Therefore, optimization of their microbiome may contribute to an improvement in reproductive outcomes in infertile women undergoing assisted reproductive technology treatments.

A range of approaches have been suggested, from basic dietary adjustments to the use of antibiotics and vaginal microbiota transplants.^(27)^ One therapeutic approach has been of particular interest, involving supplementation probiotics containing mainly Lactobacillus species to restore women's reproductive health.^(28)^ The benefit of probiotic supplementation in the treatment of bacterial vaginosis has already been evaluated in several clinical trials and literature reviews, with available evidence suggesting that probiotics favor the prevention or treatment of bacterial vaginosis.^(29–31)^ Probiotics can be administered via various routes, including vaginal and oral routes.^(32)^ Oral administration holds the advantage of convenience and higher adherence rates.^(28)^ However, despite these promising prospects, no scientific or clinical evidence supports their efficacy.^(8)^ Hence, further research should be carried out to determine their efficacy and safety.

The primary objective of this literature review is to assess the impact of probiotics rich in Lactobacillus strains on pregnancy outcomes among infertile women undergoing assisted reproductive technology. The main parameters to be examined include clinical pregnancy rates, live birth rates, miscarriage rates, and any adverse events associated with probiotic supplementation.

Ultimately, the results of this literature review may help clinical practice by providing information on the potential role of probiotics and can identify gaps in current knowledge on which further studies should focus to better understand the mechanisms underlying the impact of probiotics on fertility outcomes.

## Methods

A systematic search of the literature was conducted across the PubMed, MEDLINE, and Scopus databases using predefined search terms: ("probiotic*" AND "pregnancy" AND "infertility" AND "women"); ("probiotic*" AND "infertility" AND "women"); ("probiotic*" AND "microbiota" AND "fertility" AND "women"); ("probiotic*" AND "reproduction*" AND "pregnancy" AND "women").

- Population: Infertile women undergoing ART
- Intervention: Supplementation with probiotics
- Comparison: No probiotic supplementation
- Outcome: Biochemical and clinical pregnancy rates, live birth rates, and miscarriage rates

Following the initial search, duplicates, reviews, and other non-relevant documents were removed. Subsequently, the remaining articles underwent a two-stage screening process, initially based on title and abstract, followed by a full-text assessment. Articles lacking measurable parameters related to fertility, studies involving populations with established diseases, and unpublished studies were excluded. Additionally, reference lists of selected articles were examined to identify potentially relevant studies. A total of six articles meeting the predefined criteria were selected for inclusion in this literature review.

For each included study, relevant information was extracted, including study design, objectives, sample size, baseline characteristics of participants, probiotic strains, doses, routes, and duration of administration, as well as fertility outcomes.

## Results

### Study characteristics

Of the six studies included, two are randomized controlled trials,^(33,34)^ one is a retrospective controlled study,^(35)^ one is a prospective controlled study,^(26)^ and two are non-randomized clinical trials.^(19,36)^ One of the non-randomized clinical trials, conducted by Gilboa et al.,^(36)^ employed a pseudo-randomization approach. All the studies examined the impact of probiotic supplementation in women undergoing ART. These studies are listed in chart 1. with their summary characteristics and relevant clinical results.

**Chart 1:** Characteristics and relevant clinical results of included studies

Article	Study design	Population		Probiotic				Objective	Outcomes
		Type	Size	Route	Strain	Dose	Days		
Gilboa et al. (2005)^(36)^	Clinical Trial	Infertile patients	117	Vaginal	*Lactobacillus acidophilus, Bifidobacterium bifidum* and *Bifidobacterium longum* (3x10^9^ CFU)	2 capsules	3	To investigate the effect of probiotics on vaginal colonization and on outcome of the IVF cycle.	The probiotic did not affect the vaginal colonization of *Lactobacillus* during embryo transfer. The clinical pregnancy rate improved slightly, albeit with no statistical significance.
Iniesta et al. (2022)^(19)^	Clinical Trial	Infertile couples	14	Oral	*Lactobacillus salivarius* PS11610 (1x10^9^ CFU)	1 capsule/12 hours	180	To evaluate the effect of *L. salivarius* PS11610 on the microbial composition of urogenital tract in infertile couples with bacterial dysbiosis.	Probiotic supplementation significantly modified the urogenital microbiome composition. Additionally, slightly improved the pregnancy and delivery rates, albeit with no statistical significance.
Di Pierro et al. (2023)^(35)^	Retrospective, observational, study	Infertile patients	160	Oral	*Lactobacillus crispatus* M247 (2x10^9^ CFU)	1 sachet/day	90	To evaluate whether the oral administration of *L. crispatus* could increase pregnancy and live birth rates in women undergoing ART.	The rate of biochemical and clinical pregnancy rates, as well as live births were higher in the probiotic group, although with no statistical significance.
Tanha et al. (2023)^(33)^	Randomized, controlled trial	Infertile patients	103	Vaginal	*Lactobacillus rhamnosus* (1x10^9^ CFU)	1 suppository/day	14	To evaluate the effect of *Lactobacillus rhamnosus* on normalizing vaginal microbiome, and its potential to enhance outcomes in FET cycles.	In the Lactovag group, biochemical and clinical pregnancy rates were higher, albeit without statistical significance. The rate of pregnancy loss in the control group was fivefold in comparison with the Lactovag group.
Thanaboonyawat et al. (2023)^(34)^	Randomized, controlled trial	Infertile patients	316	Vaginal	*Lactobacillus acidophilus* KS400 (1x10^6^ CFU)	1 tablet/day	6	To compare the biochemical pregnancy rate between women using intravaginal probiotic supplementation and those with standard treatment before embryo transfer in FET cycles.	The biochemical and clinical pregnancy rates were comparable in both groups. There was a statistically significant reduction in the miscarriage rate in the study group.
Wei et al. (2024)^(26)^	Prospective crontrolled trial	Infertile patients	60	Vaginal	*Lactobacillus delbrueckii* DM8909	-	30	To investigate the impact of transvaginal *Lactobacillus* supplementation on reproductive outcomes in patients with prior failed FET cycles	Transvaginal supplementation significantly increased the clinical pregnancy rate, while the miscarriage rate showed no difference between the two groups.

CFU - colony-forming unit; IVF - in vitro fertilization; ART - assisted reproductive technology; FET - frozen embryo transfer; OR - odds ratio

### Patient characteristics

While five studies provided detailed demographic characteristics of their populations, Iniesta et al.^(19)^ only mentioned that all participants were caucasian, with an average age of 35 for women and 36 for men. When comparing baseline characteristics between control and intervention groups in these studies, no statistically significant differences were observed.

### Intervention characteristics

The probiotics administered were made up of a variety of species, either individually or in combination. The most frequently employed species was *Lactobacillus acidophilus*, which appeared in two studies.^(34,36)^ In addition, other species included *Lactobacillus rhamnosus, Lactobacillus crispatus, Lactobacillus salivarius, Lactobacillus gasseri, Lactobacillus fermentum, Bifidobacterium bifidum, Bifidobacterium longum,* and *Lactobacillus delbrueckii.* The routes of administration differed across the studies, with four employing vaginal administration and two oral administrations. Additionally, the duration of treatment varied significantly, spanning from 2 days to 6 months.

### Clinical pregnancy rate

Regarding outcomes, all included studies reported either biochemical or clinical pregnancy rates. Gilboa et al.^(36)^ evaluated the effect of intravaginal probiotic supplementation with *Lactobacillus acidophilus, Bifidobacterium bifidum,* and *Bifidobacterium longum*, on vaginal colonization after oocyte retrieval and the outcome of embryo transfer cycles. No significant differences were observed in clinical pregnancy rates with 36.0% in the study group versus 34.3% in the control group (p = 0.85).^(36)^

Iniesta et al.^(19)^ investigated the impact of supplementation with *L. salivarius PS11610* on the microbial composition of the urogenital tract in infertile couples with bacterial dysbiosis. It was revealed that supplementation was correlated with an enhanced pregnancy and delivery ratio. Specifically, the study reported that four women became pregnant (44.4%) following the intervention, two after artificial insemination and two spontaneously. Three of them successfully gave birth (33.3%) but one had an ectopic pregnancy.^(19)^

Di Pierro et al.^(35)^ concluded that the administration of a probiotic containing *Lactobacillus crispatus* M247 resulted in increased clinical pregnancy and live birth rates. In the study group, a biochemical pregnancy occurred in 23.75% compared to 17.50% in the control group, with all progressing to clinical pregnancies (OR: 1.56, 95% CI: 0.6976–3.4953). Regarding live births, the probiotic group exhibited a rate of 12.5% compared to 7.5%, yielding an odds ratio of 1.76 (95% CI: 0.608–5.103).

Tanha et al.^(33)^ found that Lactovag, containing *Lactobacillus rhamnosus*, could enhance the likelihood of pregnancy following frozen-thawed embryo transfer. In the study group, the biochemical pregnancy rate was 30.6%, while 25.0% in the control group, resulting in an odds ratio of 1.32 (95% CI: 0.53–3.30). As for clinical pregnancy, the rates were 28.6% versus 17.8%, yielding an odds ratio of 1.85 (95% CI: 0.69–4.94). Additionally, the rate of miscarriage in the control group was fivefold higher compared to the Lactovag group.^(33)^

Thanaboonyawat et al.^(34)^ investigated the pregnancy outcomes of frozen-thawed embryo transfer cycles following supplementation with *Lactobacillus acidophilus* KS400. The biochemical and clinical pregnancy rates were comparable between the study and control groups (39.9% versus 41.8% and 34.2% versus 31.7%, respectively). The implantation rate was slightly higher in the study group, 24.8% compared to 21.4% in the control group. Despite the lack of statistical significance observed (p > 0.05), a notable reduction in the miscarriage rate was observed in the probiotic group (9.5% versus 19.1%), yielding an odds ratio of 0.44 (95% CI: 0.23–0.86; p = 0.02). However, statistically significant results were observed among the subgroup undergoing blastocyst transfer. In this subgroup, the miscarriage rate was significantly reduced in the study group (8.2% versus 24.3%, p = 0.002), resulting in an odds ratio of 0.28 (95% CI: 0.12–0.65), and the live birth rate was significantly higher (35.7% versus 22.2%, p = 0.03), with an odds ratio of 1.9 (95% CI: 1.05–3.59).^(34)^

Wei et al.^(26)^ evaluated the impact of supplementation with a probiotic containing *Lactobacillus delbrueckii* DM8909 on reproductive outcomes in patients with previous failed frozen embryo transfer cycles. The supplementation led to a significant increase in the clinical pregnancy rate compared to the control group (66,7% versus 36.7%, p = 0.04), while no significant difference was observed in the miscarriage rate between the two groups (10.0% versus 13.3%, p = 0.63).^(26)^

### Microbiome parameters

Although the microbiome was not directly assessed before oocyte collection in Gilboa et al.,^(36)^ vaginal wall swabs were obtained and processed either for culture or Gram staining. Similarly, swabs for culture and Gram staining were collected again from the vagina at the time of embryo transfer, 48-72 hours post-oocyte collection. Despite intravaginal supplementation with probiotics immediately after oocyte collection, there was no observed effect on vaginal colonization by lactobacilli during embryo transfer. Furthermore, no significant association was found between the presence of lactobacilli before oocyte collection or embryo transfer and pregnancy rate.^(36)^

Iniesta et al.^(19)^ examined the bacterial composition of vaginal, glans, and semen samples from participating couples at the study's onset and after 3 and 6 months of treatment. Dysbiosis was evaluated using culture-dependent techniques. At the study's outset, all 14 couples exhibited bacterial dysbiosis. Yet, treatment with L. salivarius PS11610 resolved dysbiosis in the great majority of the couples.^(19)^

Analysis of the vaginal microbiota composition post 3 and 6 months of probiotic intake indicated a notable reduction not only in total bacterial count but also in *Staphylococcus spp., Streptococcus spp.,* Pathogens and Other potentially harmful bacterial populations (p = 0.014, p = 0.001, p = 0.0152, p = 0.003, p = 0.032, respectively). Concurrently, the proportion of Lactobacillus relative to the total bacterial count increased. Specifically, the average percentage of *Lactobacillus spp.* in vaginal samples escalated from 10% at study initiation to 38% and 61% after 3 and 6 months, respectively.^(19)^ Additionally, the microbial composition of vaginal, uterus, glans, and semen samples were characterized through 16S rRNA sequencing. Although no statistically significant changes were noted when comparing samples before and after treatment, principal component analysis plots revealed alterations in the microbiome composition of uterine samples post 6 months of probiotic therapy.^(19)^

The baseline characteristics of the two groups of women in Di Pierro et al.^(35)^ were compared by analyzing the results of vaginal-rectal swabs collected before ovarian stimulation using culture methods. The analysis revealed no significant differences between the groups.^(35)^

On the final day of assessing endometrial thickness in Thanaboonyawat et al.,^(34)^ vaginal discharge was collected, and its color, pH, and odor were noted. A wet smear was obtained, and the Amsel criteria were employed for diagnosing bacterial vaginosis. The prevalence of bacterial vaginosis was 17.7% in the study group and 14.1% in the control group, although no statistically significant differences were observed. In a post hoc subgroup analysis of 49 women diagnosed with bacterial vaginosis, the study group exhibited higher clinical pregnancy and live birth rates compared to the control group (42.3% versus 34.8% and 42.31% versus 26.09%, respectively), although these differences were not statistically significant (p = 0.59 and p = 0.23, respectively).^(34)^

Wei et al.^(26)^ found a significant correlation between vaginal microecology and pregnancy outcomes (χ^2^ = 17.344, p < 0.001). Vaginal samples were collected on the day of transfer, before the procedure, using a swab. Gram staining was utilized to evaluate bacterial density, species, and dominant bacteria. Among participants with normal vaginal microecology, 64.3% belonged to the clinical pregnancy (CP) group, 9.8% to the miscarriage (MISC) group, and 25.9% to the non-pregnant (NP) group. In contrast, individuals with dysbiosis exhibited varied proportions, with 43.2% in the CP group, 10.6% in the MISC group, and 46.2% in the NP group. Regarding microbiota dominance by lactobacilli, 60.3% belonged to the CP group, 48.7% to the MISC group, and 50.0% to the NP group (p = 0.116).^(26)^

The influence of vaginal microecology on the composition of the endometrial microbiota during frozen embryo transfer cycles was also assessed. Endometrial samples revealed a low presence of *Lactobacillus* (2.7%), with the predominant species being *Rhodococcus* (23.7%), *Pseudomonas* (4.9%), and *Achromobacter* (4.1%). However, no statistically significant differences were observed among the three groups (p = 0.09). Notably, the abundance of *Achromobacter* exhibited a positive correlation with clinical pregnancy (p = 0.0127), while the abundance of *Romboutsia, Psychrobacter, Roseiflexaceae,* and *Chryseobacterium* showed negative correlations (p = 0.013, p = 0.02661, p = 0.0275, p = 0.03203, respectively).^(26)^

The variance inflation factor analysis identified the classification of Lactobacillus as the main factor influencing the composition of the endometrial microbiota, with a value of 3.61. Employing Bray–Curtis-based dbRDA, microbial variation in endometrial samples was significantly explained not only by *Lactobacillus*-dominated vaginal microbiota (p = 0.01) and *Lactobacillus* classification (p = 0.03) but also by catalase (p = 0.008) and leukocyte esterase (p = 0.001).^(26)^

### Tolerability parameters

Only Di Pierro et al.^(35)^ evaluated the tolerability of probiotics administered. It was concluded that they were well-tolerated, with adverse events being nearly identical in both groups. These events included constipation, flatulence, bloating, gastralgia, nausea, and headaches, with similar incidence rates observed in both the probiotic group (11.25%) and the control group (12.50%).^(35)^

## Discussion

This review compiles available data concerning the impact of probiotics on pregnancy outcomes among infertile women undergoing assisted reproductive technology. While some previous reviews on this topic have explored the effects of probiotics on altering the vaginal microbiome, only one has assessed their influence on meaningful fertility outcomes, such as pregnancy rates.^(37)^

The microbiome and its role in human health is a complex area of study that, despite being extensively researched, still lacks conclusive data. Currently, there are no established clinical guidelines recommending the evaluation of the vaginal or endometrial microbiome, nor advocating empirical treatment with antibiotics or probiotics, in infertile patients undergoing fertility procedures.

Furthermore, no consensus has yet been reached on the best way to assess the intricate microbiome of the female genital tract. With the advent of next-generation sequencing techniques, several diagnostic tests like the Endometrial Microbiome Metagenomic Analysis (EMMA) test have emerged. The EMMA test classifies the endometrium into Lactobacillus-dominant and non-Lactobacillus-dominant profiles by analyzing samples taken by endometrial biopsy. In this way, it promises to offer an insight into a woman's reproductive prognosis based on the percentage of lactobacilli present.^(38)^ However, the scientific community has yet to define the threshold indicating a vaginal or endometrial microbiome associated with higher success rates in fertility treatments.

Combining the findings of the studies reviewed, various probiotics showed improvements in pregnancy rates across five studies, with only Wei et al.^(26)^ demonstrating a statistically significant increase (66.7% versus 36.7%, p = 0.04). This lack of significant associations raises questions about the true impact of probiotics.

In the study by Thanaboonyawat et al.,^(34)^ the biochemical and clinical pregnancy rates showed no significant differences between the study and control groups (39.9% versus 41.8% and 34.2% versus 31.7%, respectively). However, the implantation rate was slightly higher in the study group, reaching 24.8% compared to 21.4% in the control group.^(34)^ These results are in line with Moreno et al.,^(9)^ who showed a decreased miscarriage rate from 60 to 16.7% in the *Lactobacillus*-dominated microbiota group. Notably, a statistically significant decrease in the miscarriage rate was observed in the probiotic group (9.5% versus 19.1%).^(34)^

Consistent with previous research, Wei et al.^(26)^ reported the significance of vaginal microecological markers such as pH levels, *Lactobacillus*-dominance in the microbiome, *Lactobacillus* classification, catalase, and leukocyte esterase, on shaping the composition of the endometrial microbiota.^(26)^ These findings highlight the complex interaction between specific elements of vaginal and endometrial microecology, which collectively seem to impact reproductive outcomes.

A limitation of this review is the considerable heterogeneity among the included studies, which poses challenges in drawing definitive conclusions. There is notable diversity across various aspects, including the probiotics utilized, such as species, routes of administration, doses, and duration of treatment. To be able to draw conclusions, it's imperative to carefully assess and compare the benefits of different lactobacilli species. Each species possesses unique characteristics that exert distinct influences on the reproductive microbiome. For instance, Koedooder et al.^(39)^ suggest that while a high abundance of vaginal *Lactobacillus* appears beneficial for assisted reproductive technology outcomes, an excessive composition of >60% *L. crispatus* may not confer advantages.

When considering routes of administration, Tomusiak et al.^(40)^ proposed that administering probiotics vaginally might facilitate local colonization. However, as highlighted in the review by Blancafort and Llácer,^(41)^ many studies demonstrating favorable outcomes following probiotic treatment with *Lactobacillus* exhibit regression post-treatment, indicating potential challenges in long-term replication and colonization of exogenous *Lactobacillus* in the female tract.^(41)^ Therefore, it's crucial to evaluate whether the chosen route impacts the sustainability of its effect.

In two studies analyzed in this review, participants received prebiotics and/or antibiotics.^(33,35)^ Some researchers suggest prophylactic antibiotic therapy before in vitro fertilization procedures to enhance implantation rates. However, antibiotic therapy can disrupt the vaginal and endometrial flora, potentially reducing *Lactobacillus* levels.^(27,33)^ Consequently, prebiotics and antibiotics may have influenced the outcomes regarding the impact of probiotics on the microbiome. Compared to probiotics, the use of prophylactic antibiotics to modulate the microbiome before fertility treatments has some disadvantages, including the induction of resistance, susceptibility to recurrences, and potential adverse effects.^(42)^

This literature review offers valuable insights into the practical application of interventions targeting the vaginal microbiota. However, several critical questions remain to be clarified, prompting the need for further investigation. Understanding the full scope of *Lactobacillus*'s impact on female health and reproduction is crucial, particularly in determining whether microbiome modification correlates with improved reproductive outcomes in assisted reproductive technology. Moreover, the role of routine dysbiosis screening in fertility procedures requires clarification. Additionally, the debate over whether the emphasis should be on analyzing the endometrial microbiome or the vaginal microbiome persists, given the lack of conclusive evidence regarding the influence of the vaginal microbiome on the endometrial microbiome.

## Conclusion

With this review, it is not possible to infer the true role of probiotics in modifying the results of ART. However, the results of our review are in line with the results of other reviews that evaluated the influence of probiotics on female health and reproduction. In conclusion, further research is necessary to recommend the evaluation of the pre-ART microbiome to optimize it using probiotic treatment. Hence, new randomized control trials are suggested to evaluate the benefits of probiotics, particularly regarding the proper species, dosage, route of administration, and duration. Moreover, it is important to assess whether these effectively modulate the vaginal and endometrial microbiome and whether these changes endure over time.
